# Exploring the antioxidant, antiglycation, and anti-inflammatory potential of *Oroxylum indicum* stem bark extracts

**DOI:** 10.1371/journal.pone.0325795

**Published:** 2025-06-12

**Authors:** Soraya Rodwattanagul, Pathomwat Wongrattanakamon, Siripat Chaichit, Chuda Chittasupho, Wutigri Nimlamool, Wim E. Hennink, Siriporn Okonogi

**Affiliations:** 1 PhD Degree Program in Pharmacy, Faculty of Pharmacy, Chiang Mai University, Chiang Mai, Thailand; 2 Department of Pharmaceutical Sciences, Faculty of Pharmacy, Chiang Mai University, Chiang Mai, Thailand; 3 Center of Excellence in Pharmaceutical Nanotechnology, Chiang Mai University, Chiang Mai, Thailand; 4 Department of Pharmacology, Faculty of Medicine, Chiang Mai University, Chiang Mai, Thailand; 5 Department of Pharmaceutics, Faculty of Science, Utrecht Institute for Pharmaceutical Sciences, Utrecht University, Utrecht, The Netherlands; Bowen University, NIGERIA

## Abstract

Degenerative diseases occur when humans suffer from oxidative stress, glycation and prolonged inflammation. This study explores the antioxidant, antiglycation, and anti-inflammatory effects of extracts of *Oroxylum indicum*, a plant used in traditional medicines in Asia. Several extracts from its stem bark were obtained using hexane, ethyl acetate, and ethanol as extraction solvents. The extracts were analyzed using high-performance liquid chromatography (HPLC). The HPLC chromatograms showed that the different *O. indicum* extracts contained three major flavonoid compounds, namely baicalein, chrysin, and oroxylin A, as well as a phenolic compound, *p*-coumaric acid. The total phenolic content (TPC) and total flavonoid content (TFC) of the extracts were also determined. The ethyl acetate fractionated extract (EAFE) possessed the highest TPC (172 ± 8 mg/g extract) and TFC (147 ± 1 mg/g extract), several times higher than those of crude ethanol extract, ethanol fractionated extract (EFE) and hexane fractionated extract (HFE), respectively. According to the highest levels of TPC and TFC, EAFE showed the highest antioxidant activity with Trolox equivalent antioxidant activity of 9.7 ± 0.1 mM/mg and *β*-carotene bleaching inhibition of 79.5 ± 1.1%. The activities for the natural antioxidant quercetin were 3.1 ± 0.1 mM/mg and 88.7 ± 0.1%, respectively. EAFE showed the highest antiglycation activity using a bovine serum albumin-methylglyoxal assay with 89.1 ± 0.7% inhibition. These findings indicate that TPC and TFC are the determining factors for the antioxidant and antiglycation activities of the extracts. An *in silico* analysis suggested that the anti-inflammatory activity of the extracts is due to the inhibition of toll-like receptor 4 (TLR4) activity by direct binding of the bioactive compounds to the TLR4 protein. Our findings provide scientific support for the use of *O. indicum* in traditional medicine and demonstrate that EAFE has potential in mitigating oxidative stress, glycation, and inflammation.

## Introduction

Degenerative diseases among which diabetes, atherosclerosis and other cardiovascular conditions, chronic kidney failure, neurodegenerative conditions, and cancer can cause disability, mortality and morbidity especially in the elderly population [1] Protein glycation [2,3], oxidative stress [4], and prolonged inflammation [5] are contributing factors that increase the severity of these diseases. Glycation is a non-enzymatic adduct formation between the carbonyl group of reducing sugars and free amino groups of biological molecules, including proteins, lipids, and nucleic acids [6,7]. Advanced glycation end-products (AGEs), which are formed in the last stage of the process [8], can cause alteration of the structure and function of extracellular matrix proteins such as collagen, intracellular proteins such as intermediate filament, cytoskeleton proteins, enzymes, DNA, and lipids [9,10]. It has been shown in many studies that AGEs can generate reactive oxygen species (ROS) via complex biochemical mechanisms [11,12] while the formed ROS in turn accelerate the rate of AGEs formation [9,13]. The excess generated ROS can lead to oxidative stress, which is the imbalance between reactive species and an antioxidants [4]. Furthermore, ROS can stimulate nuclear transcription factor-kappa B (NF-κB) [14] to generate pro-inflammatory cytokines such as tumor necrosis factor-α (TNF-α), interleukin-6 (IL-6), and interleukin-1β (IL-1β) which all play critical roles in inflammation [15–17].

Currently, natural products from plants have proved to be potential sources of new agents able to counteract several diseases [18–20]. However, several studies focused only on one or two biological activities of plant extracts for the treatment of a certain disease. The search for plant extracts having multiple biological activities, including antioxidation, antiglycation, and anti-inflammation, is therefore a challenge for the prevention and the possibility for the treatment of degenerative diseases. *Oroxylum indicum*, a medium-sized tree belonging to the family Bignoniaceae, is a typical plant found in South and Southeast Asian countries including Sri Lanka, India, China, Malaysia, and Thailand. Different parts of this plant have been used as traditional medicines for the treatment of various conditions such as cancer, fever, gastric ulcer, arthritis, and diarrhea [21]. It was also previously reported that the ethanol extract of *O. indicum* stem bark showed wound-healing activity in the deep dermal excision wounds of mice [22]. Moreover, the dichloromethane extracts of stem bark and root bark of this plant provided antimicrobial activity against both gram-positive and negative bacteria as well as yeast [23]. Previous investigations reported that extracts of different parts of the plant contain several groups of secondary metabolites, including flavonoids, alkaloids, terpenoids, and tannins [24,25]. Although the biological activities of these extracts were reported, it was not investigated whether they were caused by their antioxidant, antiglycation, and/or anti-inflammatory activities.

The present study aimed to get insight into these different biological activities of the *O. indicum* extracts. To this end, several extracts using different extracting techniques and solvents to obtain the crude and fractionated extracts of *O. indicum* stem bark were prepared. The obtained extracts were comparatively investigated for antioxidant, antiglycation, and anti-inflammatory activities. The chemical constituents of the extracts were analyzed using high-performance liquid chromatography (HPLC). Finally, an *in silico* study was employed to get insight into the possible molecular mechanism of the anti-inflammatory effect of the extracts.

## Materials and methods

### Materials

2,2’-Azinobis-(3-ethylbenzothiazoline-6-sulphonic acid) (ABTS), gallic acid, *β*-carotene, linoleic acid, bovine serum albumin (BSA), methylglyoxal (MGO), aminoguanidine, lipopolysaccharide (LPS) from *Escherichia coli* (serotype 0111:B4), *p*-coumaric acid, quercetin, chrysin, baicalein, oroxylin A, and polysorbate 80 were from Sigma-Aldrich (St. Louis, MO, USA). Folin-Ciocalteu reagent, sodium carbonate, potassium persulphate, trifluoroacetic acid, and fetal bovine serum were from Merck (Darmstadt, Germany). Aluminum chloride was from Ajax Finechem (Sydney, Australia). Hexane, ethyl acetate, ethanol, acetonitrile (HPLC grade), chloroform, and dimethyl sulfoxide (DMSO) were from RCI Labscan Limited (Bangkok, Thailand). AlamarBlue cell viability reagent was from Invitrogen (Merelbeke, Belgium). Human monocytic (THP-1) cells were obtained from the American Type Culture Collection (ATCC, Manassas, VA, USA) (ATCC Cat# TIB-202, RRID:CVCL_0006). Complete Roswell Park Memorial Institute (RPMI) 1640 medium, penicillin, streptomycin, and 2-mercaptoethanol were from Gibco BRL Life Technologies (Gaithersburg, MD, USA).

### Preparation of *O. indicum* extracts

A fresh stem bark of *O. indicum* was collected from Chiang Mai, a province in northern Thailand, in August 2018. This plant can be cultivated and collected without the need for approval from environmental authorities as it is not on Thailand list of prohibited woods. The plant was identified by comparison with a voucher specimen number 005224 in the botanical herbarium of the Faculty of Pharmacy, Chiang Mai University, Chiang Mai, Thailand. The plant sample was washed with water and cut into small pieces and then dried at 50°C for 24 h. The dried plant was subsequently ground into a fine powder and kept in an airtight container at room temperature and protected from light until use.

For preparation of the ethanolic crude extract (ECE), the dried sample powder (about 50 g) was macerated with 500 mL of 95% ethanol at room temperature for 48 h. Then, the macerated mixture was filtered through Whatman No.1 filter paper (GE HealthCare Technologies, Chicago, IL, USA). The residue collected after filtration was further macerated with 500 mL of 95% ethanol and filtered in the same manner two more times. The filtrates from the three macerations were combined and evaporated under reduced pressure using a rotary evaporator (N-1000, Eyela, Tokyo, Japan) until the solvent was completely removed (the weight of the extract did not further decrease in time). The obtained ECE was stored at 4°C until use.

For preparation of the fractionated extracts, the dried plant powder (around 50 g) was first macerated with 500 mL of hexane. The follow up procedure was the same as for the preparation of the ECE. The residue from the hexane maceration was dried at room temperature to remove residual hexane, and the obtained dried powder was subsequently macerated with ethyl acetate in the same manner as with hexane. Finally, the dried residue was macerated with 95% ethanol in the same manner as described for hexane and ethyl acetate. The filtrates from each solvent were pooled and subsequently evaporated under vacuum using a rotary evaporator. The obtained fractionated extracts from hexane (HFE), ethyl acetate (EAFE), and ethanol (EFE) were stored at 4°C until use.

### Determination of total phenolic and flavonoid contents of the extracts

The total phenolic content (TPC) of *O. indicum* extracts was determined using the Folin-Ciocalteu assay as previously described [26] with minor modifications. Briefly, the extract was dissolved in DMSO to obtain a stock solution of 1 mg/mL. An aliquot of 20 µL of this stock solution was mixed with 45 µL of Folin-Ciocalteu reagent and incubated for 3 min. Then, 135 µL of 20 mg/mL sodium carbonate solution was added, and the mixture was incubated in the dark for 1 h at room temperature. Subsequently, the absorbance was measured at 750 nm using a microplate reader (EZ Read 2000, Biochrom, Cambridge, UK). Gallic acid (10–500 µg/mL in DMSO) was used for calibration. The TPC is expressed as mg of gallic acid equivalent (GAE) in 1 g of the extract.

The total flavonoid content (TFC) of the extracts was determined using aluminum chloride colorimetric assay as previously described [27] with some minor modifications. Briefly, the extract was dissolved in DMSO to obtain a stock solution of 1 mg/mL. An aliquot of 50 µL of this stock solution was mixed with 10 µL of 10% aluminum chloride aqueous solution. Subsequently, 150 µL of absolute ethanol was added, followed by the addition of 10 µL of 1 M sodium acetate aqueous solution. Subsequently, the mixture was incubated in the dark for 40 min at room temperature. Then, the absorbance at 415 nm was measured using a microplate reader (EZ Read 2000, Biochrom). Quercetin (30–100 µg/mL in DMSO) was used for calibration. The TFC is expressed as mg of quercetin equivalent (QE) in 1 g of the extract.

### HPLC analysis of the extracts

The crude and fractionated extracts of *O. indicum* were analyzed using HPLC (Prominence-i LC-2030, Shimadzu, Kyoto, Japan) connected with a reversed-phase C18 column, 4 mm i.d. × 250 mm (Eurospher II, Knauer, Berlin, Germany). The separation was performed using gradient elution. Mixtures of 0.1% trifluoroacetic acid in water (A) and acetonitrile (B) with different ratios were used as gradient eluents. The gradient program started with a 65:35 ratio of A to B and was maintained for 5 min. Then, the ratio of eluents A to B was changed to 35:65 for 10–15 min. After that, the composition of A to B eluents was changed back to 65:35 at 16 min and was maintained for 5 min. The extracts dissolved in acetonitrile (5 µL, 1 mg/mL) were injected with an eluent flow rate of 1 mL/min. The eluents were monitored with a UV detector at a wavelength of 270 nm. For qualitative analysis, four reference compounds, baicalein, chrysin, oroxylin A, and *p*-coumaric acid (the concentration of each compound was 0.1 mg/mL), were used as markers.

For quantification analysis, the chemical constituents in crude and fractionated extracts of *O. indicum* were determined using the external calibration method. Calibration curves of each reference compound were performed using a series of reference compound solutions. The limit of detection (LOD) and limit of quantification (LOQ) of each reference compound were calculated from the following equations.


LOD = 3.3σ/S

LOQ = 10σ/S

Where, σ is the standard deviation of the y-intercept of the calibration curves and *S* is the slope of the calibration curve.

The percentage recovery of each standard compound spiked into different extracts was also examined to ensure the accuracy of the method. A standard compound (0.01 mg/mL) was added to the extract (0.1 mg/mL), and the amount of the standard compound in the resulting mixture was then analyzed. The percentage recovery was calculated using the following equation.


Recovery (%) = (amount mixture – amount sample)/amount spiked compound × 100%

### Determination of antioxidant activity of the extracts

Two standard methods, namely the ABTS free radical scavenging and *β*-carotene bleaching (BCB) assays, were used for the determination of the antioxidant activity of the extracts.

The ABTS assay was performed according to a method previously described [28] with minor modifications. Briefly, ABTS free radicals were generated by mixing 8 mL of 7 mM ABTS aqueous solution with 12 mL of 2.45 mM potassium persulfate aqueous solution and subsequently incubating in the dark for 16 h. Then, DMSO was added to the ABTS free radical solution to obtain an absorbance of ~0.9 at 750 nm. The extract was dissolved in DMSO to obtain the stock solution of 0.5 mg/mL. Next, an aliquot of 20 µL of this stock solution was mixed with 180 µL of the ABTS free radical solution and incubated in the dark for 5 min at room temperature. Subsequently, the absorbance at 750 nm was measured using a microplate reader (EZ Read 2000, Biochrom). Quercetin (0.5 mg/mL in DMSO) was used as a positive standard. Trolox (50–500 µM in DMSO) was used for calibration. The results are expressed as mM of Trolox equivalent antioxidant capacity (TEAC) of 1 mg of the extract.

The BCB assay was performed according to a method previously described [29] with some modifications. Briefly, 1 mL of *β*-carotene solution (stock solution of 20 mg/mL in chloroform) was pipetted into a round bottom flask containing the mixture of 20 mg of linoleic acid and 200 mg of polysorbate 80. Then, chloroform was removed using a rotary evaporator (N-1000, Eyela) under reduced pressure at 50°C for 10 min, and 50 mL of oxygenated water was subsequently introduced in the flask with vigorous agitation to obtain an emulsion. Next, an aliquot of 20 µL of the extract solution (stock solution of 1 mg/mL in DMSO) was mixed with 180 µL of the obtained emulsion. The absorbance at 450 nm of the mixture was measured using a microplate reader (EZ Read 2000, Biochrom). Then, the mixture was further incubated in the dark for 120 min at room temperature, and the absorbance at 450 nm was subsequently measured. Quercetin at 1 mg/mL in DMSO was used as a positive standard, and DMSO was used as a negative control. The results are expressed as percentage of inhibition, which is calculated using the following equation.


$$Inhibition\;of\;\beta -carotene\;bleaching\;(\;\%)\;=\;[(C_{T0}\;–\;C_{T120})\;-\;(S_{T0}\;–\;S_{T120})/(C_{T0}\;–\;C_{T120})]\;\times \;100\;\%$$

Here, S_T0_ and S_T120_ are the absorbance of the sample at time 0 and 120 min, respectively and C_T0_ and C_T120_ are the absorbance of negative control at time 0 and 120 min, respectively.

### Determination of antiglycation activity of the extracts

The antiglycation activity of the extracts was investigated by determining the inhibitory effect on AGEs formation using BSA-MGO assay, which represents an intermediate stage of protein glycation [30–32]. The reaction between BSA and MGO results in glycation of BSA, and the formed AGEs, vesperlysines-like structures, can be detected by spectrofluorometry [30].

Stock solutions of 50 mg/mL BSA (with 0.02% sodium benzoate as a preservative), the extract at 1 mg/mL in DMSO, 50 mM phosphate buffer saline (PBS, pH 7.4), and MGO (0.01 mg/mL, 150 mM) were prepared. The reaction mixtures were prepared as described in Table 1. Sample mixtures without MGO were used as sample blanks. The mixture without the extract was used as a control, and the mixture without the extract and MGO was used as a control blank. Then, the mixtures were incubated at 45°C for 7 days. Subsequently, the fluorescence intensity was measured using a microplate reader (SpectraMax^®^ M3, Molecular Devices, San Jose, CA, USA) at excitation and emission wavelengths of 370 and 440 nm, respectively. Aminoguanidine at 1 mg/mL was used as a positive standard. The results are expressed as a percentage of AGEs inhibition, which is calculated using the following equation.

**Table 1:** Compositions of the reaction mixtures used in the BSA-MGO assay.

Reaction mixtures	Stock solution volume (µL)			
	Extracts (10 mg/mL)	MGO (0.01 mg/mL)	BSA (50 mg/mL)	PBS (50 mM)
Control	–	100	200	700
Control blank	–	–	200	800
Sample	100	100	200	600
Sample blank	100	–	200	700


$$Inhibition\;of\;AGEs\;formation\;(\;\%)\;=\;[(C-C_{B})\;-\;(S–S_{B})/(C-C_{B})]\;\times \;100\;\%$$

Here, C and C_B_ are the fluorescent intensities of the control and its blank, respectively. S and S_B_ are the fluorescent intensities of the samples and their blanks, respectively.

### *In vitro* anti-inflammatory study of the extracts

#### Cell cultures.

According to ATCC recommendations, the THP-1 cells were cultured in complete RPMI 1640 medium supplemented with 10%FBS, antibiotics (100 U/mL penicillin and 100 µg/mL streptomycin), and 0.05 mM 2-mercaptoethanol at 37°C with 5% CO_2_ for 72 h before performing the experiments.

#### Cytotoxic effect of the extracts on THP-1 cells.

The cytotoxicity of the extracts on THP-1 cells was investigated to select non-toxic concentrations that resulted in cell viability above 80% [33]. AlamarBlue cell viability assay was performed according to the manufacturer’s protocol (Invitrogen, Merelbeke, Belgium) with slight modifications. Briefly, 100 μL of THP-1 cells suspended in the culture medium at a concentration of 1 × 10^6^ cells/mL were seeded into a 96-well plate and incubated overnight at 37°C with 5% CO_2_. The serial dilution of *O. indicum* extract in cell culture medium was prepared by mixing 5 μL of 20 mg/mL extract stock solution in DMSO with 395 μL of cell culture medium, and then adding more medium to obtain two-fold dilutions. Subsequently, 100 μL of the various concentrations of the extracts were added to the wells containing cells to achieve final concentrations ranging from 0 to 125 µg/mL, followed by incubation for 24 h. After 24 h, the cells completely adhered to the well surfaces, and 100 μL of the culture medium was removed. Then, 10 μL of 10-fold alamarBlue cell viability reagent was pipetted into the wells and incubated in the dark at 37°C for 3 h. Subsequently, the optical density was determined at 562 nm and 600 nm as a reference wavelength using a microplate reader (AccuReader M965, Metertech, Taipei, Taiwan). The results are expressed as a percentage of cell viability, which is calculated using the following equation.


$$Cell\;viability\;(\;\%)\;=\;({OD}_{treated}/{OD}_{untreated})\;\times \;100\;\%$$

Where OD is optical density, and the percentage of cell viability of the untreated cells is defined as 100%.

#### Effect of the extracts on pro-inflammatory cytokine production.

The effect of the two non-cytotoxic concentrations (0.5 and 1 µg/mL) of the extracts on IL-6 production from inflamed THP-1 cells was determined using an enzyme-linked immunosorbent assay (ELISA) [34,35]. The THP-1 cells suspended in culture medium were seeded into a 24-well plate (0.5 × 10^6^ cells/well) and incubated overnight at 37°C with 5% CO_2_. Then, the cells were incubated with the extracts at 0.5 and 1 μg/mL and 0.02% DMSO for 4 h before further incubation with 0.1 μg/mL LPS for 24 h. Subsequently, the cell supernatants were analyzed for secreted IL-6 using the human IL-6 ELISA kit (catalog no. 430504) according to the manufacturer’s instructions (BioLegend, San Diego, CA, USA). The optical density of the samples was measured using a microplate reader (AccuReader M965, Metertech) at 450 nm and 562 nm as a reference wavelength. The results are expressed as a percentage of IL-6 secretion calculated using the following equation.


$$Secreted\;IL-6\;(\;\%)\;=\;(IL-6_{sample}/IL-6_{LPS})\;\times \;100\;\%$$

The IL-6_sample_ represents its concentration in the cell culture medium of the cells pretreated with the extracts and triggered with LPS, and IL-6_LPS_ represents the concentrations of the secreted IL-6 from the cells treated with LPS only. The IL-6 secretion from the cells treated with LPS only is defined as 100%.

### *In silico* anti-inflammatory study

#### Construction of ligand structures.

The 3D molecular structures of the docking ligands, baicalein (CID: 5281605), chrysin (CID: 5281607), oroxylin A (CID: 5320315), and *p*-coumaric acid (CID: 637542), were downloaded from PubChem [36]. The molecular energies of the structures were minimized using ChemBio3D ultra 11.0, and the Gasteiger partial atomic charges were added to the structures using AutoDockTools.

#### Molecular docking.

AutoDock program was employed to construct each ligand–TLR4 model via the Lamarckian genetic algorithm (LGA). The 126 × 126 × 126 Å spaced 0.375 Å grid was designed to cover the defined binding site of TLR4 (PDB:3FXI) obtained from Discovery Studio Client 2.5 through the ‘Define and Edit Binding Site’ tool. The calculation of 200 GA runs was conducted. For conformational clustering, the 1.0 Å tolerance value of root-mean-square deviation (RMSD) was applied. Regarding the selection of the candidate conformation from each binding model, a ligand conformation exhibiting the lowest docking score (kcal/mol) was collected.

#### Molecular dynamics (MD) simulation.

AMBER18 with the PMEMD dynamics engine was used to perform the MD simulations of toll-like receptor 4 (TLR4)–ligand complexes. The force field ff03.r1 [37] was applied to the docking models of TLR4 and the ligands, baicalein, chrysin, oroxylin A, and *p*-coumaric acid, from the molecular docking covering the entire simulated systems. Antechamber was used to generate force field parameters of the ligands. TLeap was used to generate topology and coordinate parameters for each simulated complex. The water model of TIP3P as the truncated octahedral periodic box was used to solvate the complex. Nineteen sodium ions were put into the model to neutralize its charge. Minimizing, 20 picoseconds (ps) heating and 60 ps equilibrating stages were run for the whole system. The NVT method was applied for the last two stages. Finally, the NVT method with 300 Kelvin and 1 atm was employed for the 50,000 ps production run to generate a dynamic structure of the ligand–TLR4 complex. A stability parameter of each dynamic model of ligand–TLR4 interaction was shown by RMSD. The RMSD plot of each ligand–TLR4 interaction model was calculated. A parameter regarding binding free energy was shown as the Molecular Mechanics Generalized Born Surface Area (MM-GBSA) scoring using representative 250 frames from the 50,000 ps trajectory through the MMPBSA.py script.

### Statistical analysis

The experiments were carried out in triplicate. The results are expressed as mean ± standard deviation (SD). A one-way analysis of variance (ANOVA) with Tukey’s post-hoc multiple comparison tests was calculated to determine statistical differences between means using SPSS statistical software package v.17.0. For the *in vitro* anti-inflammatory study, the statistical difference of IL-6 secretion levels was determined using independent t-test. The significant threshold (P-value, *p*) was set at < 0.05.

## Results and discussion

In this study, the antioxidant, antiglycation, and anti-inflammatory activities of *O. indicum* stem bark extracts were investigated. The crude extract, ECE, and the three fractionated extracts, HFE, EAFE, and EFE, were subjected to chemical analysis prior to the biological activity investigation.

### Chemical analysis of *O. indicum* extracts

**Fig 1:**
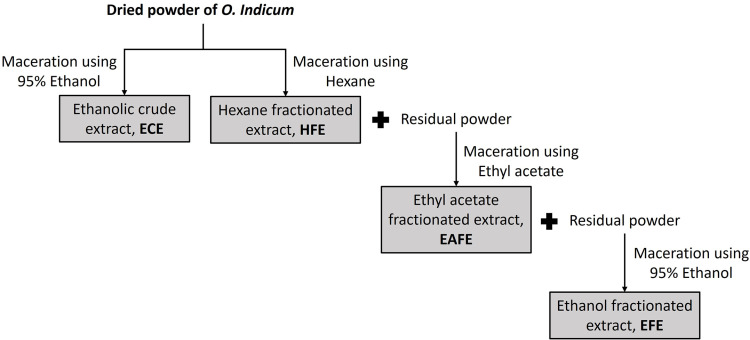
Chart of the preparation of *O. indicum* extracts.

According to the different extracting techniques and solvents shown in Fig 1, the yield, TPC, and TFC of each extract was different as shown in Table 2. It was found that the TPC of the different extracts expressed as GAE values ranged from 42 to 172 mg/g extract while their TFC expressed as QE values ranged from 17 to 147 mg/g extract. EAFE showed the highest GAE value of 172 ± 8 mg/g extract and the highest QE value of 147 ± 1 mg/g extract, which were significantly higher than other extracts (*p* < 0.05). These results indicate that *O. indicum* extracts indeed contained phenolics and flavonoids in different. It is known that phenolic compounds are important plant secondary metabolites that are composed of an aromatic ring bearing at least one hydroxyl group. Among these phenolic compounds, flavonoids present as a major group in the form of aglycone, glycoside and methylated derivatives [38]. Flavonoids as well as many other phenolic components have been reported to have several effective biological activities such as antioxidant, anticancer, anti-inflammation and skin protection from UV radiation [39–42]. The results of the TPC and TFC assays, therefore, demonstrate that *O. indicum* extracts indeed contained phenolics and flavonoids in different amounts.

**Table 2:** The yield, TPC as GAE values, and TFC as QE values of *O. indicum* extracts.

Extracts	Yield* (%)	GAE* (mg/g extract)	QE* (mg/g extract)
ECE	5.9 ± 2.1^a^	86 ± 5^b^	64 ± 1^b^
HFE	0.3 ± 0.2^c^	42 ± 3^c^	17 ± 1^d^
EAFE	1.0 ± 0.5^b,c^	172 ± 8^a^	147 ± 1^a^
EFE	5.1 ± 0.6^a,b^	53 ± 2^c^	31 ± 1^c^

* Data represents mean ± SD (n = 3). Statistical analysis of data within the same column was analyzed by a one-way analysis of variance (ANOVA) with Tukey’s post-hoc multiple comparison test. Lowercase letters indicate significant differences (*p* < 0.05).

**Fig 2:**
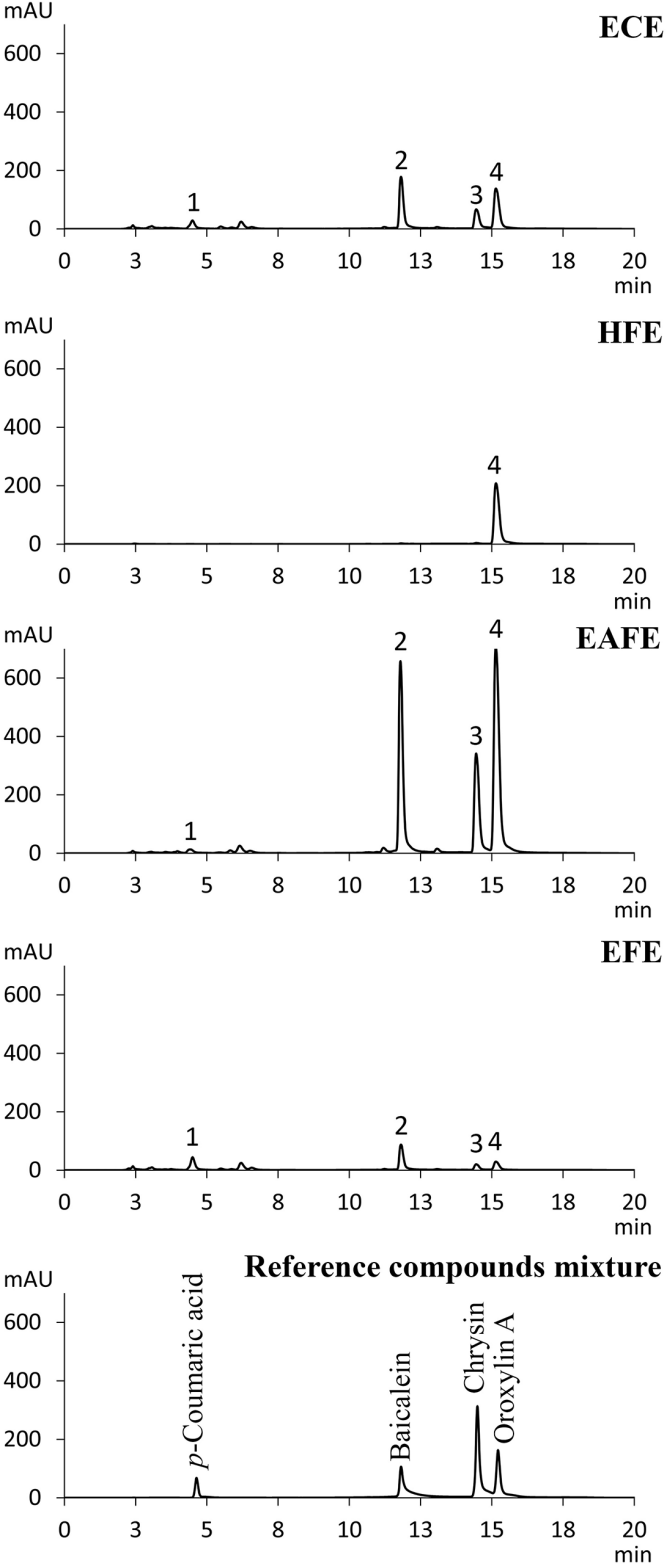
HPLC chromatograms of *O. indicum* extracts and a mixture of reference compounds.

The different extracts were analyzed using HPLC. Separation was done with a C18 reversed-phase column and detection was at 270 nm. This wavelength was selected since the UV spectra of the different extracts showed the highest absorption at this wavelength as shown in S1 Fig of the Supporting information. It is noted that in the extracts of many medicinal plants, the strong antioxidant quercetin is present in high amounts [43]. Since quercetin has a strong maximum in its UV spectrum at 380 nm, it is concluded from the spectra shown in S1 Fig that in the *O. indicum* stem bark extracts this compound is likely not present in high concentrations. This is in line with publications of Wei et al. and Chalermwongkul et al. that quercetin has been found in the seed but not in the stem bark of this plant [44,45]. Fig 2 shows the HPLC chromatograms of the different extracts as well as those of three reference flavonoid compounds (baicalein, chrysin, oroxylin A) and one reference phenolic compound (*p*-coumaric acid). The chemical structures of these reference compounds are shown in S2 Fig of the Supporting information. Peaks 1, 2, 3, and 4 of the extracts as shown in Figs 2A-D, respectively, exhibited retention times that correspond to the peaks that of *p*-coumaric acid, baicalein, chrysin, and oroxylin A which were 4.4, 11.8, 14.4, and 15.1 min, respectively as shown in Fig 2E. These results confirmed previous reports which provide that these four compounds present in stem bark of *O. indicum* [21,46,47].

The amounts of chemical constituents in *O. indicum* extracts were determined using calibration curves for each reference compound, and the results are shown in Table 3. The LOD and LOQ of the reference compounds, as shown in Table 3, indicate the sensitivity of each compound analyzed using the current method. Quantitative analysis was conducted at concentrations higher than the LOD and LOQ. The percentage recovery of the reference compounds spiked into the extracts is presented in Table 4, ranging from 96% to 103%, which falls within the acceptable range of 95–105% [48].

**Table 3:** The calibration curves of reference compounds and limit of detection.

Compound	RT (min)	Calibration equation*	Correlation factor, R^2^ **	LOD (µg/mL)	LOQ (µg/mL)
*p*-Coumaric acid	4.4	y = 10816x + 5.5589	0.9999	0.09	0.27
Baicalein	11.8	y = 19388x + 12.128	1.0000	0.76	2.31
Chrysin	14.4	y = 24833x + 36.474	1.0000	0.54	1.64
Oroxylin A	15.1	y = 25502x + 96.636	0.9999	0.52	1.59

RT, retention time; LOD = Limit of detection, LOQ = Limit of quantification; * y = peak area, x = concentration of reference compounds (mg/mL), n = 3; ** R^2 ^= correlation coefficient of six data points.

**Table 4:** The percentage recovery of reference compounds spiked in *O. indicum* extracts.

Extracts	Recovery (%)			
	*p*-Coumaric acid	Baicalein	Chrysin	Oroxylin A
ECE	100.2 ± 0.5	101.6 ± 0.1	96.7 ± 1.9	99.8 ± 1.4
HFE	99.6 ± 0.6	101.1 ± 0.9	98.1 ± 2.5	102.9 ± 1.3
EAFE	100.9 ± 0.3	99.1 ± 0.8	98.6 ± 1.4	97.6 ± 0.2
EFE	101.8 ± 0.1	97.3 ± 0.2	99.5 ± 0.1	99.5 ± 0.2

Data represents mean ± SD (n = 3).

The LOD, LOQ, and percentage recovery results confirm the accuracy of the quantitative analysis presented in Table 5. The chromatograms and these results show that the total area under the curve (AUC) of the peaks of the EAFE was by far the highest, which agrees with the strongest observed absorption of this extract at 270 nm (S1 Fig).

**Table 5:** Quantitative analysis of chemical constituents of *O. indicum* extracts.

AUC and Content	Compound	Extracts			
		ECE	HFE	EAFE	EFE
Total AUC	–	4,789	2,829	20,411	1,976
AUC (%)	*p*-Coumaric acid	7	ND	1	26
	Baicalein	38	Tr	33	45
	Chrysin	17	Tr	20	11
	Oroxylin A	38	98	46	18
Content* (mg/mg extract)	*p*-Coumaric acid	0.03	ND	0.01	0.05
	Baicalein	0.09	Tr	0.35	0.05
	Chrysin	0.03	Tr	0.16	0.01
	Oroxylin A	0.07	0.11	0.37	0.01

AUC, area under the curve; ND, not detectable; Tr, trace; *, the contents of the extracts were determined using calibration curves of the reference compounds.

Obviously, ethyl acetate, a dipolar aprotic solvent [49], has the highest solubility for the flavonoid compounds present in the extracts as previously observed for the dihydroxyflavone chrysin [50]. Thus, the highest contents of all three flavonoids were extracted by ethyl acetate (Table 5). On the other hand, *p*-coumaric acid was better extracted with ethanol, a polar protic solvent, as also observed in a previous publication [51]. Interestingly, in HFE only oroxylin A was detected at a content of 0.11 mg/mg extract. Baicalein, chrysin and oroxylin A are flavones with slightly different substitution moieties, while *p-*coumaric acid is a phenolic acid derivative. Oroxylin A provides the highest lipophilicity among the four compounds. Thus, oroxylin A therefore is extracted in higher amount than other mentioned compounds by non-polar solvents such as hexane [52]. It can be noted that HFE might contain other unidentified bioactive compounds soluble in hexane, a non-polar solvent, such as sterols, alkaloids, terpenoids, and fats [53,54].

### Antioxidant activity

Several methods are available to evaluate antioxidant activity of plant extracts. In the present study, the ABTS and BCB assays were used. Free radicals are a major cause of the propagation stage of oxidation processes. Compounds that are able to scavenge free radicals can therefore inhibit the spreading of oxidation. The ABTS assay is an excellent method for screening of well-defined compounds as well as plant extracts for their free radical-scavenging ability [55]. The BCB assay is also one of the standard methods for testing antioxidant activity used in the field of food chemistry [56]. The principle of this method is based on the discoloration of the yellowish color of a *β*-carotene solution caused by free radicals generated by autoxidation of linoleic acid. The potential antioxidant can cause the retardation of this discoloration by competing with *β*-carotene in reacting with the free radicals [57,58]. The results of the ABTS and BCB assays with the different plant extracts are presented in Table 6. It was found that the EAFE had the highest TEAC value of 9.7 ± 0.1 mM/mg extract, followed by ECE, HFE, and EFE with TEAC values of 5.7 ± 0.1, 4.8 ± 0.1, 4.2 ± 0.2 mM/mg extract, respectively. The TEAC value of quercetin was 3.1 ± 0.1 mM/mg compound, demonstrating that the different *O. indicum* extracts had a stronger scavenging activity than the positive standard. The stronger antioxidant effects of the plant extract than the golden standard quercetin as found in the current study was also found in other publications [59,60].

**Table 6:** Antioxidant and antiglycation activities of *O. indicum* extracts.

Extracts	Antioxidant activity		Antiglycation activity
	ABTS (TEAC, mM/mg)	BCB (% inhibition)	BSA-MGO (% inhibition)
ECE	5.7 ± 0.1^ab^	64.8 ± 2.8^c^	78.7 ± 0.4^c^
HFE	4.8 ± 0.1^b^	56.0 ± 1.5 ^cd^	54.2 ± 0.5^d^
EAFE	9.7 ± 0.1^a^	79.5 ± 1.1^b^	89.1 ± 0.7^a^
EFE	4.2 ± 0.2^b^	55.3 ± 3.3^d^	55.9 ± 1.1^d^
Quercetin	3.1 ± 0.1^b^	88.6 ± 0.1^a^	–
Aminoguanidine	–	–	85.1 ± 0.4^b^

Data represents mean ± SD (n = 3). Statistical analysis of data within the same column was analyzed by a one-way analysis of variance (ANOVA) with Tukey’s post-hoc multiple comparison test. Lowercase letters indicate significant differences (*p* < 0.05).

This result also shows that EAFE also possessed the highest inhibitory effect using the BCB assay with *β*-carotene bleaching inhibition of 79.5 ± 1.1%, followed by ECE, HFE, and EFE with the inhibition of 64.8 ± 2.8, 56.0 ± 1.5, 55.3 ± 3.3%, respectively. This indicates that all *O. indicum* extracts provided excellent inhibitory activity (over 50% inhibition) slightly lower, particularly for EAFE, than quercetin, the positive standard (*β*-carotene bleaching inhibition of 88.6 ± 0.1%). Taken together, the results demonstrate that EAFE possessed the highest level of TPC and TFC which in turn resulted in the highest antioxidant activity using both antioxidant assays. Our results are in good agreement with a study of Zhang et al. [61] who found a positive linear correlation between antioxidant activity and both TPC and TFC of some medicinal plant extracts. Interestingly, HFE, a non-polar extract, also provided high inhibition of *β*-carotene bleaching (more than 50%). Siramon and Ohtani have reported that very hydrophobic compounds, including *p*-cymene, *g*-terpinene, and 1,8-cineole, showed very low DPPH radical scavenging activity, but showed high inhibition of *β*-carotene bleaching [62]. The *β*-carotene bleaching assay is commonly used for determining the antioxidant activity of non-polar compounds. It is noted that the more polar compounds present in the EFE preferentially partition in the aqueous phase and to a lower extent in the linoleic acid phase of the emulsion of the BCB reaction mixture and, hence, less effective in protecting linoleic acid from oxidation which is named in the literature as the “polar paradox” [63]. Taken together, the results demonstrate that the *O. indicum* extracts provide antioxidant activity via various mechanisms.

### Antiglycation activity

As mentioned in the introduction, AGEs are harmful products that are formed in the final stage of glycation. AGEs are divided into two types, namely (A) fluorescent and crosslinked AGEs and (B) non-fluorescent and non-crosslinked AGEs [31]. The antiglycation activity of *O. indicum* extracts was evaluated using the BSA-MGO assay. MGO is a sugar derivative with a highly reactive carbonyl group that is formed in the intermediate stage of glycation [64]. MGO can react with particularly lysine and arginine residues in proteins, as BSA present in the reaction mixture of the BSA-MGO assay, to yield the formation of AGEs [65]. The results, as shown in Table 6, reveal that the EAFE possessed the highest percentage of AGEs inhibition (89.1 ± 0.7%), followed by ECE, EFE, and HFE (78.7 ± 0.4, 55.9 ± 1.1, and 54.2 ± 0.5%), respectively. These results indicate that the AGEs inhibitory activity of EAFE was slightly higher than that of aminoguanidine, a positive control (85.1 ± 0.4%). This stronger AGE inhibitory activity EAFE than the positive control was also reported in studies of other plant extracts [66,67]. The antiglycation activity of different extracts follows the same trend as their TPC and TFC values as reported in Table 2. Other studies also reported a positive correlation between antiglycation activity and total phenolic and flavonoid contents [68–70] and between antiglycation and antioxidant activities of plant extracts [70]. Our results thus show that the phenolic and flavonoid compounds in *O. indicum* extracts play a pivotal role in inhibiting AGEs production, products from reaction between proteins and MGO.

### Anti-inflammatory activity

#### *In vitro* anti-inflammatory study.

**Fig 3:**
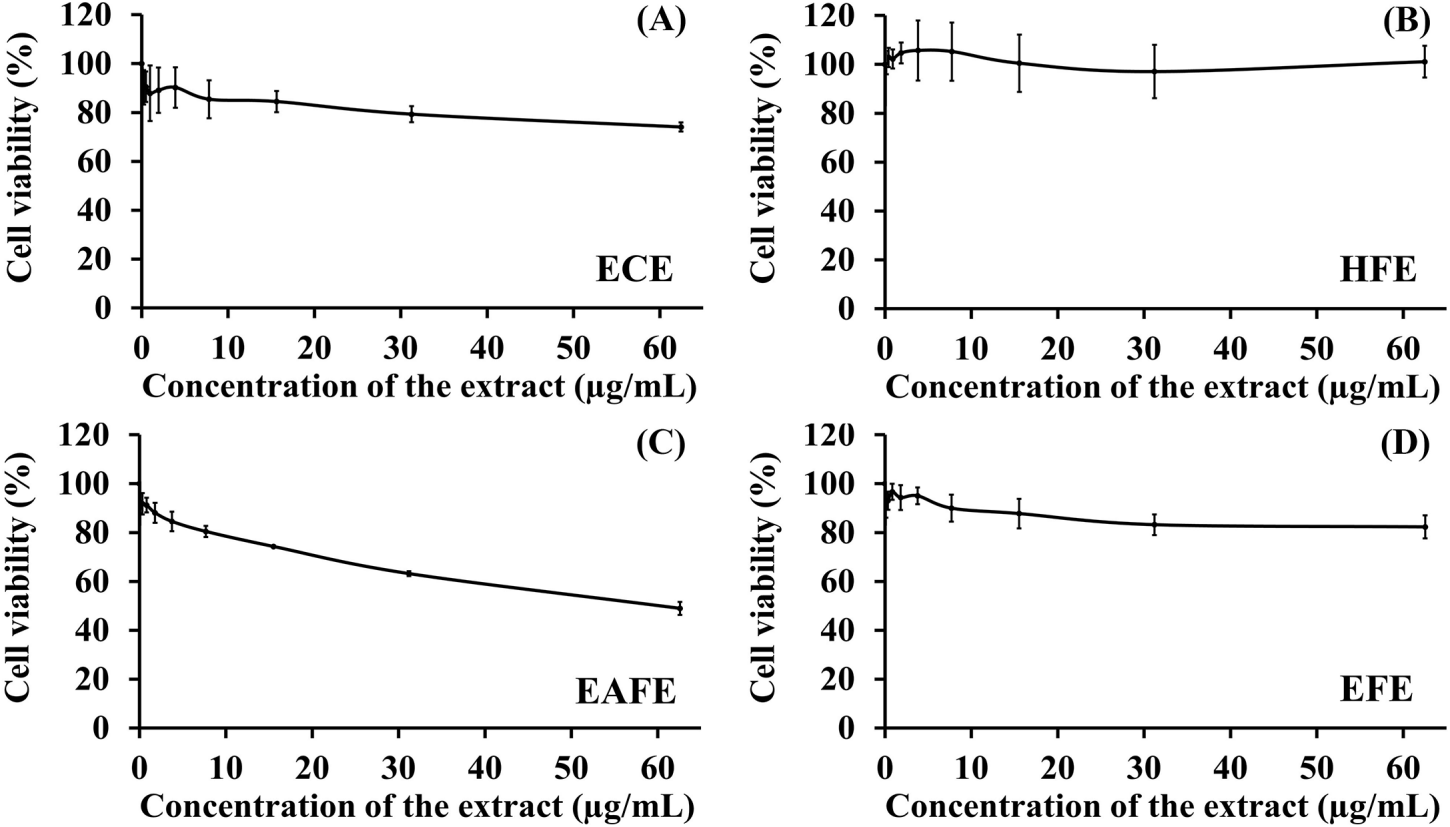
Cell viability of THP-1 cells after incubation with the different extracts. Data represents mean ± SD (n = 3).

Fig 3 shows the cytotoxic effect of the extracts against THP-1 cells. The results show that different extracts exhibited different cytotoxic effects. The HFE, EFE and ECE were non-toxic to the cells (cell viability above 80%) at concentrations up to 63 μg/mL in the cell culture medium and upon incubation for 24 h at 37°C. On the other hand, the EAFE showed toxic effects on the cells in a concentration-dependent manner with an IC_50_ value of ~60 µg/mL. It is remarked that the observed cytotoxic effects are not due to residual solvents since the samples were extensively dried to an extent that no further weight loss was observed. In line with our results, Matsuo et al. reported that some flavonoids showed considerable cytotoxicity on normal human cells at relatively high concentrations and in a dose-dependent manner. They suggested that these flavones and flavonols exert cytotoxicity through increasing intracellular ROS levels [71]. Indeed, HPLC analysis (Fig 2 and Table 5) showed the highest concentration of the flavones baicalein, chrysin and oroxylin A in EAFE. It is finally noted that for the anti-inflammatory study, concentrations of the extracts far below that of the toxic concentrations (0.5 and 1 μg/mL) were selected.

IL-6 is a pro-inflammatory cytokine that plays a pivotal role in both acute and chronic responses. Elevated IL-6 levels are associated with various inflammatory diseases such as rheumatoid arthritis, Crohn’s disease, cardiovascular diseases, different types of cancer, and diabetes [72,73]. It was reported that IL-6 levels were significantly elevated in patients with age-related diseases compared to the controls, while the levels of TNF and IL-1β did not show a consistent association with these diseases [74]. Therefore, the effect of the extracts on IL-6 secretion by THP-1 cells exposed to LPS was studied and the results are depicted in Table 7. The result shows that IL-6 was not detected in the medium of the unstimulated cells (detection limit 9.5 pg/mL), whereas upon stimulation with LPS this cytokine was secreted at a concentration of 413 ± 2 pg/mL. DMSO, the solvent used for dissolving the extracts and up to a concentration of 0.02% v/v, did not affect the IL-6 secretion. The result also shows that the *O. indicum* extracts at a concentration of 1 µg/mL significantly inhibited IL-6 secretion of LPS-stimulated cells (*p* < 0.05). At this concentration, HCE inhibited IL-6 secretion by 38 ± 9%, followed by ECE, EAFE, and EFE, which inhibited IL-6 secretion by 24 ± 4%, 20 ± 9%, and 17 ± 7%, respectively. The HFE tends to exhibit higher anti-inflammatory effects than other extracts. There are some compounds in a non-polar extract from *O. indicum* stem bark that have been reported to present anti-inflammatory activities, such as lapachol and *β*-sitosterol [23]. Our findings are also in line with the results from another study that the hexane extract of *Echium amoenum* flowers provided good anti-inflammatory activity compared to ethyl acetate and dichloromethane extracts [75].

**Table 7:** IL-6 secretion of LPS-stimulated THP-1 cells after incubation with the extracts.

Medium	Extract concentration (µg/mL)	IL-6 secretion (%)
RPMI 1640	N/A	<detection limit (9.5 pg/mL)
RPMI 1640 plus LPS	N/A	100
RPMI 1640 plus LPS and 0.02% DMSO	N/A	95 ± 8
RPMI 1640 plus LPS and ECE	0.5	75 ± 7*
	1	76 ± 4*
RPMI 1640 plus LPS and HFE	0.5	78 ± 6*
	1	62 ± 9*
RPMI 1640 plus LPS and EAFE	0.5	91 ± 7
	1	80 ± 9*
RPMI 1640 plus LPS and EFE	0.5	95 ± 8
	1	83 ± 7*

N/A, not applicable; Data represents mean ± SD (n = 3). Significant differences in comparison with LPS treated group are indicated by * (*p *< 0.05).

#### *In silico* anti-inflammatory study.

**Fig 4:**
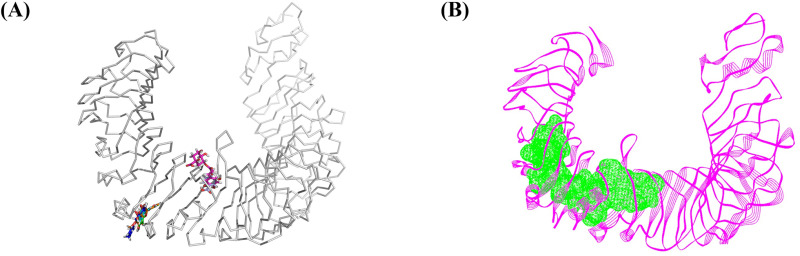
Molecular structure of TLR4. (A) The binding locations of the investigated ligands, namely baicalein (green), chrysin (blue), oroxylin A (orange) and *p*-coumaric acid (cyan) on TLR4 (gray), (B) The identified ligand-binding site (green) is shown on the structure of TLR4 (magenta).

An *in silico* study was employed to evaluate the anti-inflammatory activity of baicalein, chrysin, oroxylin A, and *p*-coumaric acid present in the extracts of *O. indicum* stem bark (Fig 2 and Table 5). The molecular docking between the compounds and TLR4, a cell surface receptor that plays a key role in initiating inflammatory signaling induced by LPS [76], was performed. The direct binding of the compounds to TLR4 can interrupt the formation of the complex of LPS–myeloid differentiation protein-2 (MD-2)–TLR4. Hence, TLR4 activity is inhibited and the inflammatory signaling is suppressed [77]. The docking poses of the compounds on TLR4 are shown in Fig 4. Baicalein, chrysin, oroxylin A, and *p*-coumaric acid indeed bind to the binding site of TLR4 as shown in Fig 4A, which is the TLR4–MD-2 interface and its adjacent area [36] was identified by Discovery Studio Client 2.5 (Fig 4B).

Binding affinity and binding stability between the ligands and TLR4 were calculated employing the MM-GBSA method. Binding free energy values for these compounds interacting with the TLR4 surface exhibited the following tendency: oroxylin A > baicalein > chrysin > *p*-coumaric acid as shown in Table 8.

**Table 8:** Interaction of the compounds and TLR4 expressed as binding free energy values.

Compounds	Binding free energy (MM-GBSA energy; kcal/mol)
Baicalein	−33.0089
Chrysin	−24.5696
Oroxylin A	−33.5571
*p*-Coumaric acid	−15.7947

**Fig 5:**
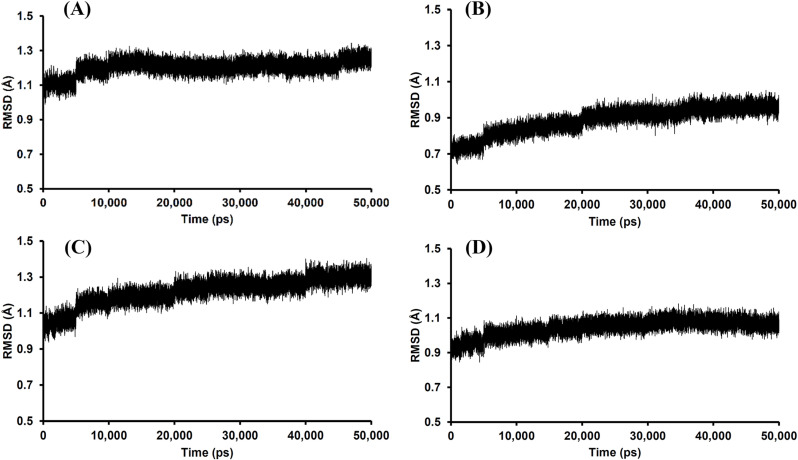
The RMSD plots throughout the 50,000 ps simulation of the ligand–TLR4 coordinates from the (A) oroxylin A, (B) baicalein, (C) chrysin, and (D) *p*-coumaric acid models.

The RMSD plots of each compound–TLR4 interaction model which can predict the structural stability are shown in Fig 5. The convergent behavior of the oroxylin A–TLR4 complex was confirmed by its RMSD plot as shown in Fig 5A, which was stable in the range of 1.2–1.3 Å after 10,000 ps compared to the baicalein, chrysin and *p*-coumaric acid–TLR4 complex, which RMSD had been increasing by time (0–50,000 ps) as shown in Figs 5B-D. These results demonstrate that oroxylin A exhibited the highest affinity towards TLR4 (indicated by the high MM-GBSA value) and provided a stable oroxylin A–TLR4 complex (the highest stability RMSD pattern) compared to other compounds. The computational results support the results of the cellular experiments, that the inhibitory effects of the extracts on IL-6 secretion upon stimulation with LPS might be due to the direct binding of flavonoid and phenolic compounds to TLR4 on THP-1 cells.

## Conclusion

The present study shows the antioxidation, antiglycation, and anti-inflammation effects of *O. indicum* stem bark extracts. The three flavonoid compounds, namely baicalein, chrysin, and oroxylin A, as well as a phenolic compound, *p*-coumaric acid, are present in the extracts. The *in silico* study supports the presence of anti-inflammatory potential of the extracts, which is very likely due to the inhibition of TLR4 activity by the interaction of bioactive compounds investigated (especially oroxylin A) and TLR4. Our findings demonstrate that *O. indicum* stem bark extracts are an interesting potential natural source for preventing degenerative diseases, especially EAFE and HFE. However, the identity of other non-polar compounds in HFE should be further investigated.

## Supporting information

S1 Fig UV spectra of four *O. indicum* stem bark extracts. (TIF)

S2 Fig Chemical structures of the different reference compounds used in this study. (TIF)

S1 File Minimal datasets. (PDF)
