# Supplementary figures and images for: Exploring the antioxidant, antiglycation, and anti-inflammatory potential of Oroxylum indicum stem bark extracts
Source: PLoS One. 2025 Jun 12;20(6):e0325795. doi: 10.1371/journal.pone.0325795 (PMC12161542; doi:10.1371/journal.pone.0325795)

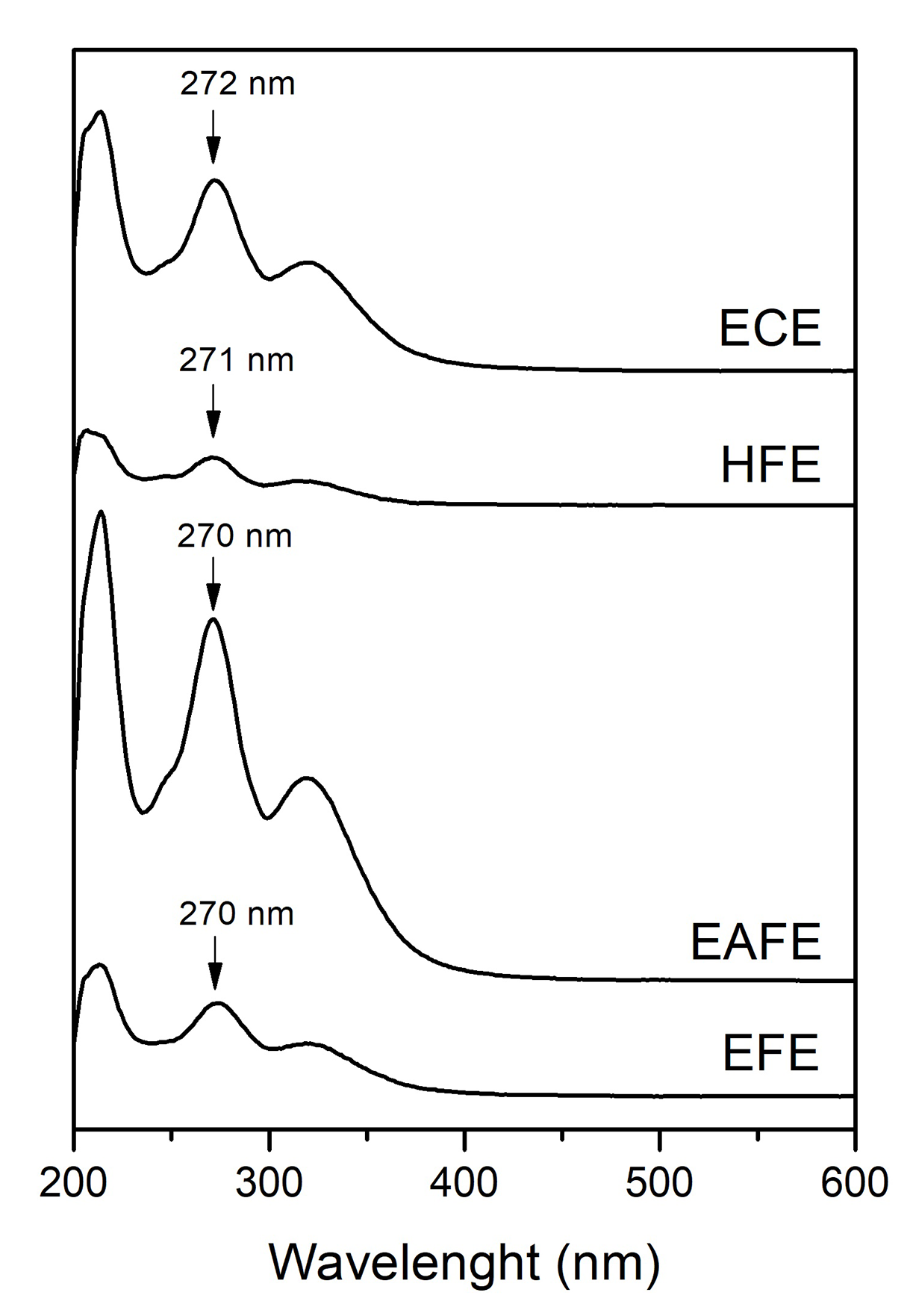

Supplement: S1 Fig — (TIF) [file pone.0325795.s001.tif]

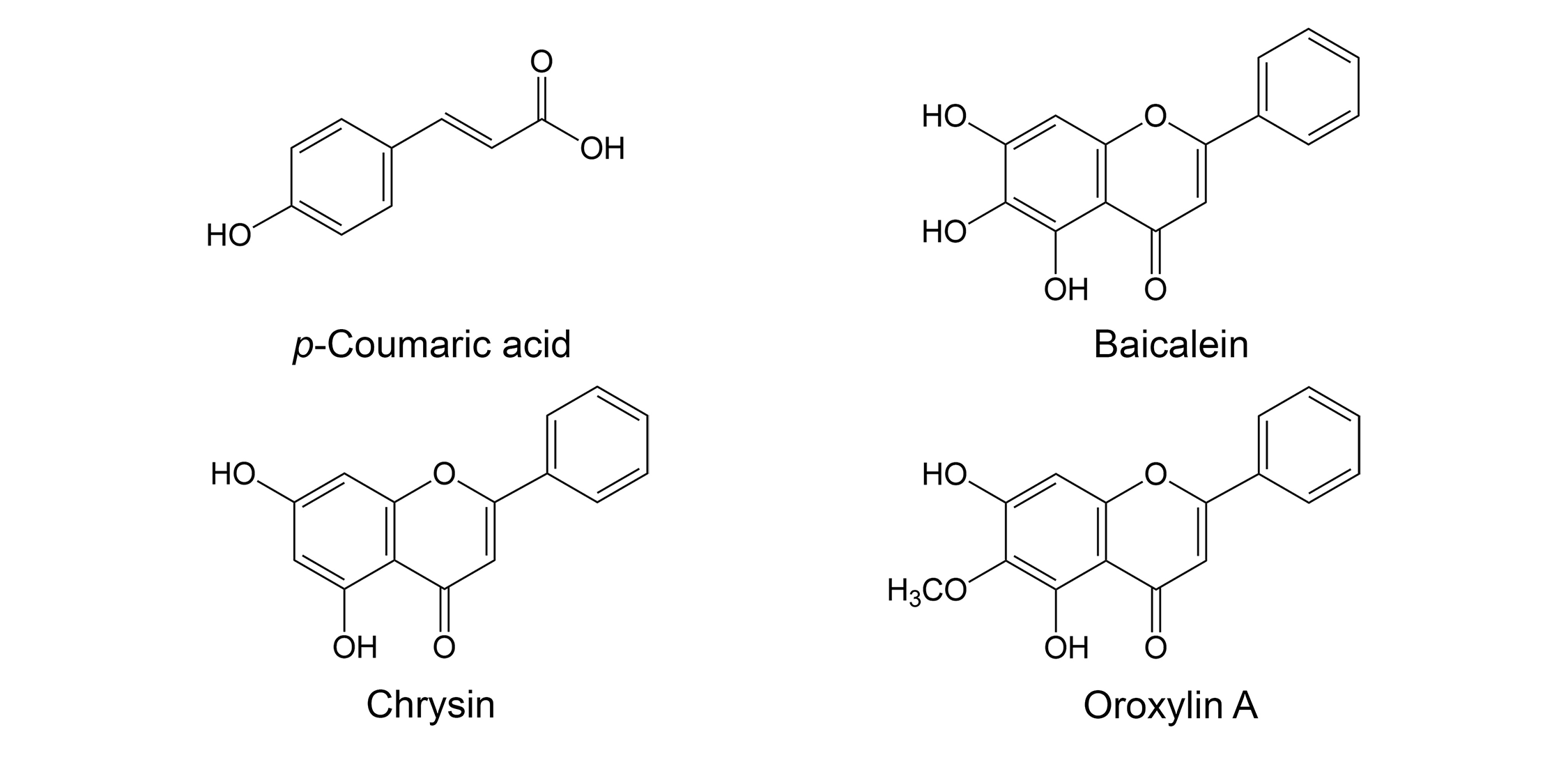

Supplement: S2 Fig — (TIF) [file pone.0325795.s002.tif]
