# Supplementary material for: Exploring the antioxidant, antiglycation, and anti-inflammatory potential of Oroxylum indicum stem bark extracts
Source: PLoS One. 2025 Jun 12;20(6):e0325795. doi: 10.1371/journal.pone.0325795 (PMC12161542; doi:10.1371/journal.pone.0325795)
Supplement: S1 File — (PDF) [file pone.0325795.s003.pdf]

S1 File. Minimal datasets

Table 1.1. The data of extraction yields

**Extract**	**%Yield**				
	n1	n2	n3	Average	SD
**ECE**	3.89	8.01	5.92	5.94	2.06
**HFE**	0.21	0.25	0.54	0.33	0.18
**EAFE**	1.64	0.78	0.81	1.08	0.49
**EFE**	4.68	4.81	5.8	5.10	0.61

Table 1.2. The data of total phenolic content

**Extract**	**GAE value (mg/g extract)**				
	n1	n2	n3	Average	SD
**ECE**	83.20	83.20	92.95	86.453	5.63
**HFE**	45.60	38.64	42.53	42.256	3.49
**EAFE**	175.13	179.30	162.59	172.340	8.70
**EFE**	56.46	51.73	52.84	53.677	2.48

Table 1.3. The data of total flavonoid content

**Extract**	**QE value (mg/g extract)**				
	n1	n2	n3	Average	SD
**ECE**	65.23	65.23	64.37	64.94	0.50
**HFE**	17.70	17.82	17.08	17.53	0.39
**EAFE**	146.90	148.26	146.54	147.23	0.91
**EFE**	31.33	31.82	31.33	31.50	0.28

Table 1.4. The values used to create the calibration curve of *p*-coumaric acid

Concentration (mg/mL)	AUC				
	n1	n2	n3	Average	SD
**0.003**	36.21	36.22	36.34	36.26	0.07
**0.006**	71.41	72.75	70.27	71.48	1.24
**0.013**	142.21	141.67	143.14	142.34	0.74
**0.025**	281.21	281.51	280.72	281.15	0.40
**0.050**	545.49	544.53	545.02	545.01	0.48
**0.100**	1086.52	1086.08	1087.16	1086.59	0.54

Table 1.5. The values used to create the calibration curve of baicalein

Concentration (mg/mL)	AUC				
	n1	n2	n3	Average	SD
**0.016**	302.87	323.49	305.52	310.63	11.22
**0.031**	623.13	619.22	626.63	622.99	3.71
**0.063**	1207.93	1213.28	1218.19	1213.13	5.13
**0.125**	2429.79	2416.85	2437.29	2427.98	10.34
**0.250**	4890.26	4886.76	4893.38	4890.13	3.31
**0.500**	9691.95	9662.89	9726.95	9693.93	32.08

Table 1.6. The values used to create the calibration curve of chrysin

Concentration (mg/mL)	AUC				
	n1	n2	n3	Average	SD
**0.008**	210.39	209.089	211.641	210.37	1.28
**0.016**	427.78	415.12	440.25	427.72	12.57
**0.031**	807.65	801.96	813.33	807.65	5.69
**0.063**	1602.22	1595.55	1608.71	1602.16	6.58
**0.125**	3161.51	3162.59	3160.45	3161.52	1.07
**0.250**	6232.32	6213.63	6249.51	6231.82	17.95

Table 1.7. The values used to create the calibration curve of oroxylin A

Concentration (mg/mL)	AUC				
	n1	n2	n3	Average	SD
**0.016**	397.54	394.42	400.79	397.58	3.19
**0.031**	845.65	842.32	848.85	845.61	3.27
**0.063**	1627.74	1631.55	1623.82	1627.70	3.87
**0.125**	3321.68	3321.36	3319.59	3320.88	1.13
**0.250**	6603.65	6597.88	6610.89	6604.14	6.52
**0.500**	13005.08	13038.27	12920.78	12988.04	60.57
**1.000**	25545.59	25487.95	25458.56	25497.37	44.27

Table 1.8. The values from calibration curve equations used for calculating LOD and LOQ of reference compounds

Compound	Y-intercept			SD of y-intercept, σ	Slope, *S*	LOD (µg/mL)	LOQ (µg/mL)
	n1	n2	n3				
***p*-Coumaric acid**	5.55	5.85	5.26	0.29	10816	0.09	0.27
**Baicalein**	9.29	17.47	10.24	4.47	19388	0.76	2.31
**Chrysin**	36.44	32.43	40.56	4.07	24833	0.54	1.64
**Oroxylin A**	91.99	99.40	98.53	4.05	25502	0.52	1.59

Table 1.9. The data of the percentage recovery of reference compounds spiked in the extracts

Extract	Compound	Recovery (%)				
		n1	n2	n3	Average	SD
**ECE**	***p*-Coumaric acid**	100.71	99.75	100.23	100.23	0.48
	**Baicalein**	101.76	101.53	101.64	101.64	0.11
	**Chrysin**	98.69	94.77	96.76	96.74	1.96
	**Oroxylin A**	98.44	101.18	99.81	99.81	1.37
**HFE**	***p*-Coumaric acid**	99.00	100.20	99.60	99.60	0.60
	**Baicalein**	102.00	100.01	100.99	101.00	0.99
	**Chrysin**	95.57	100.51	98.01	98.03	2.47

	**Oroxylin A**	101.67	104.25	102.96	102.96	1.29
**EAFE**	***p*-Coumaric acid**	100.61	101.22	100.91	100.91	0.30
	**Baicalein**	98.29	99.81	99.06	99.06	0.76
	**Chrysin**	97.21	99.99	98.58	98.59	1.39
	**Oroxylin A**	97.39	97.71	97.55	97.55	0.16
**EFE**	***p*-Coumaric acid**	101.76	101.93	101.85	101.85	0.09
	**Baicalein**	97.52	97.04	97.27	97.28	0.24
	**Chrysin**	99.58	99.49	99.54	99.54	0.04
	**Oroxylin A**	99.34	99.64	99.49	99.49	0.15

Table 1.10. The AUC values from peaks of chemical constituents in *O. indicum* extracts used for quantitative analysis

**AUC**	**Compound**	**Extract**			
		**ECE**	**HFE**	**EAFE**	**EFE**
**Total AUC**	-	4,789.65	2,829.05	20,411.40	1,976.69
**AUC**	***p*-Coumaric acid**	348.43	ND	159.93	505.77
	**Baicalein**	1,807.62	Tr	6,727.10	887.07
	**Chrysin**	816.22	Tr	4,089.62	224.63
	**Oroxylin A**	1,817.39	2,784.31	9,434.75	359.22

Table 1.11. The data from ABTS assay

**Extract**	**TEAC value (mM/mg)**				
	n1	n2	n3	Average	SD
**ECE**	5.75	5.92	5.62	5.76	0.15
**HFE**	4.79	4.84	4.86	4.83	0.04
**EAFE**	9.67	9.59	9.84	9.70	0.13
**EFE**	4.09	4.04	4.51	4.22	0.26
**Quercetin**	3.02	3.05	2.99	3.02	0.03

Table 1.12. The data from BCB assay

Extract	% Inhibition				
	n1	n2	n3	Average	SD
**ECE**	67.18	65.64	61.70	64.84	2.82
**HFE**	57.54	54.42	56.03	56.00	1.56
**EAFE**	79.89	80.36	78.30	79.52	1.08
**EFE**	52.09	55.26	58.72	55.36	3.32
**Quercetin**	88.69	88.64	88.65	88.66	0.02

Table 1.13. The data from BSA-MGO assay

Extract	% Inhibition				
	n1	n2	n3	Average	SD
**ECE**	78.28	79.15	78.78	78.74	0.44
**HFE**	54.34	54.72	53.67	54.24	0.53
**EAFE**	88.63	88.67	89.91	89.07	0.73
**EFE**	56.40	54.67	56.64	55.90	1.07
**Aminoguanidine**	84.61	85.21	85.46	85.09	0.44

Table 1.14. The data of cell viability of THP-1 after incubation with ECE

Concentration (μg/mL)	Cell viability (%)				
	n1	n2	n3	Average	SD
**0.00**	100.00	100.00	100.00	100.00	0.00
**0.24**	82.11	94.55	94.18	90.28	7.08
**0.49**	83.78	96.04	91.70	90.51	6.21
**0.98**	74.76	94.16	94.64	87.85	11.34
**1.95**	78.43	95.31	93.49	89.08	9.27
**3.91**	80.66	94.48	95.49	90.21	8.28
**7.81**	76.40	89.57	90.19	85.39	7.79
**15.63**	79.88	85.00	88.45	84.44	4.31
**31.25**	75.60	80.31	81.91	79.27	3.28
**62.50**	75.54	71.94	74.58	74.02	1.86

Table 1.15. The data of cell viability of THP-1 after incubation with HFE

**Concentration (µg/mL)**	**Cell viability (%)**				
	n1	n2	n3	Average	SD
**0.00**	100.00	100.00	100.00	100.00	0.00
**0.24**	106.18	98.92	97.04	100.71	4.83
**0.49**	107.43	100.81	100.34	102.86	3.97
**0.98**	106.51	98.95	101.20	102.22	3.89
**1.95**	109.16	100.66	104.15	104.66	4.27
**3.91**	119.60	101.40	96.11	105.70	12.32
**7.81**	119.02	99.19	97.57	105.26	11.94
**15.63**	114.05	93.34	94.05	100.48	11.75
**31.25**	109.65	90.18	91.32	97.05	10.93
**62.50**	108.53	98.25	96.48	101.09	6.51

Table 1.16. The data of cell viability of THP-1 after incubation with EAFE

**Concentration (µg/mL)**	**Cell viability (%)**				
	n1	n2	n3	Average	SD
**0.00**	100.00	100.00	100.00	100.00	0.00
**0.24**	88.46	89.50	93.38	90.45	2.59
**0.49**	89.05	89.59	96.86	91.83	4.36
**0.98**	91.44	88.16	94.15	91.25	3.00
**1.95**	87.83	84.20	92.36	88.13	4.08
**3.91**	85.74	80.16	87.84	84.58	3.97
**7.81**	81.87	77.85	81.69	80.47	2.27
**15.63**	73.93	74.68	74.32	74.31	0.37
**31.25**	63.04	62.32	64.28	63.21	0.99
**62.50**	50.94	46.00	50.04	48.99	2.63

Table 1.17. The data of cell viability of THP-1 after incubation with EFE

Concentration (µg/mL)	Cell viability (%)				
	n1	n2	n3	Average	SD
**0.00**	100.00	100.00	100.00	100.00	0.00
**0.24**	89.89	97.27	86.97	91.38	5.31
**0.49**	93.87	96.25	88.98	93.04	3.71
**0.98**	95.47	100.41	94.12	96.67	3.31
**1.95**	98.39	95.95	88.59	94.31	5.10
**3.91**	98.09	95.58	91.38	95.02	3.39
**7.81**	93.18	93.13	83.55	89.95	5.54
**15.63**	91.97	90.46	80.79	87.74	6.06
**31.25**	86.47	84.67	78.43	83.19	4.22
**62.50**	83.66	86.13	77.04	82.28	4.70

Table 1.18. The data of IL-6 secretion of LPS-stimulated THP-1 cells after incubation with the extracts

Medium	Extract concentration (µg/mL)	IL-6 secretion (%)				
		n1	n2	n3	Average	SD
**RPMI 1640**	N/A	< detection limit (9.5 pg/mL)				
**RPMI 1640 plus LPS**	N/A	100				
**RPMI 1640 plus LPS and 0.02% DMSO**	N/A	90.56	104.81	89.79	95.05	8.46
**RPMI 1640 plus LPS and ECE**	0.5	79.22	79.74	67.34	75.43	7.01
	1	80.70	71.90	74.78	75.79	4.49
**RPMI 1640 plus LPS and HFE**	0.5	81.43	81.88	70.19	77.83	6.62
	1	50.10	66.48	68.04	61.54	9.94

**RPMI 1640 plus LPS and EAFE**	0.5	99.36	87.64	86.29	91.10	7.19
	1	91.28	74.75	74.04	80.02	9.76
**RPMI 1640 plus LPS and EFE**	0.5	99.16	90.48	94.99	94.88	4.34
	1	89.91	74.61	83.73	82.75	7.70
